# Human snRNA genes use polyadenylation factors to promote efficient transcription termination

**DOI:** 10.1093/nar/gkt892

**Published:** 2013-10-04

**Authors:** Dawn O’Reilly, Olga V. Kuznetsova, Clelia Laitem, Justyna Zaborowska, Martin Dienstbier, Shona Murphy

**Affiliations:** ^1^Sir William Dunn School of Pathology, University of Oxford, South Parks Road, Oxford OX1 3RE, UK and ^2^CGAT, MRC Functional Genomics Unit, Department of Physiology, Anatomy and Genetics, University of Oxford, South Parks Road, Oxford OX1 3PT, UK

## Abstract

RNA polymerase II transcribes both protein coding and non-coding RNA genes and, in yeast, different mechanisms terminate transcription of the two gene types. Transcription termination of mRNA genes is intricately coupled to cleavage and polyadenylation, whereas transcription of small nucleolar (sno)/small nuclear (sn)RNA genes is terminated by the RNA-binding proteins Nrd1, Nab3 and Sen1. The existence of an Nrd1-like pathway in humans has not yet been demonstrated. Using the U1 and U2 genes as models, we show that human snRNA genes are more similar to mRNA genes than yeast snRNA genes with respect to termination. The Integrator complex substitutes for the mRNA cleavage and polyadenylation specificity factor complex to promote cleavage and couple snRNA 3′-end processing with termination. Moreover, members of the associated with Pta1 (APT) and cleavage factor I/II complexes function as transcription terminators for human snRNA genes with little, if any, role in snRNA 3′-end processing. The gene-specific factor, proximal sequence element-binding transcription factor (PTF), helps clear the U1 and U2 genes of nucleosomes, which provides an easy passage for pol II, and the negative elongation factor facilitates termination at the end of the genes where nucleosome levels increase. Thus, human snRNA genes may use chromatin structure as an additional mechanism to promote efficient transcription termination *in vivo*.

## INTRODUCTION

Transcription termination is the final stage of the transcription cycle when the RNA polymerase stops transcribing and disengages from the DNA template. Although little is known about the mechanism(s) by which RNA polymerase II (pol II) is released, several factors are implicated in the termination process (1–4). In yeast, pol II uses two distinct pathways for termination of transcription of mRNA and small nuclear (sn)/small nucleolar (sno)RNA genes, and the factors involved often recognize specific modifications of pol II (5–7). The C-terminal domain (CTD) of the large subunit of pol II comprises multiple repeated heptapeptides (52 repeats in mammalian cells) of the consensus sequence Tyr1-Ser2-Pro3-Thr4-Ser5-Pro6-Ser7, which is dynamically modified by phosphorylation (Tyr, Thr and Ser) and proline isomerization during the transcription cycle (8,9). Ser5 phosphorylation predominates early in the cycle, whereas Ser2 phosphorylation is higher towards the end of protein-coding genes. Consequently, as pol II transcribes the poly A signal, a component of the cleavage and polyadenylation complex, Pcf11, associates with the phospho-Ser2 form of the polymerase. As Pcf11 also associates with RNA, it is thought to tether the polymerase to the transcript, exerting enough force to reduce its processivity, which leads to termination of transcription (10–12). In addition, 3′-end processing factors, including cleavage and polyadenylation specificity factor (CPSF), cleavage stimulatory factor (CstF) and members of the Associated with Pta1 (APT) complex, including Ssu72, promote cleavage of the RNA at the poly A site, creating an entry site for the 5′–3′ exonuclease, Rat1. Rat1 degrades the downstream RNA and this is thought to help dissociate the elongation complex (13).

The second termination pathway is mainly used during transcription of sno/snRNA genes and occurs independently of RNA cleavage. Three key players have been identified; two RNA-binding proteins, Nrd1 and Nab3, and the DNA/RNA helicase, Sen1 (14–17). Nrd1 interacts specifically with the Ser5P form of pol II and is thought to assist termination of transcription relatively close to the transcription start site (TSS) (15,18,19). As for mRNA genes, termination of transcription of sno/snRNA genes is tightly coupled to 3′-end processing (20). Members of the TRAMP (Trf4/Air2/Mtr4p polyadenylation) complex aid the association of Nrd1 with the nascent RNA and also oligoadenylate the pre-sno/snRNAs to facilitate processing by the nuclear exosome complex.

In mammals, transcription of protein-coding genes is terminated by a mechanism analogous to that described in yeast (21,22), whereas the complements of cis- and trans-acting factors directing termination of the pol II-transcribed sno/snRNA genes are currently not known. Human cells do not express an equivalent of Nab3, and the Sen1 homologue, Senataxin, does not appear to participate in snRNA gene transcription termination (23). Mammalian snoRNA genes are encoded within introns of pre-mRNAs and non-coding pre-mRNA-like transcripts and are co-transcriptionally processed, bypassing a requirement for transcription termination control (24). Moreover, 3′-end processing of human snRNAs is not directed by the nuclear exosome complex but instead relies on a multisubunit complex, known as Integrator, which is not conserved in lower eukaryotes (8,25). Interestingly, components of this complex share homology with factors involved in mRNA 3′-end processing, suggesting that human snRNA genes may use a distinct transcription termination pathway that has more in common with mRNA genes than yeast snRNA genes.

Our results suggest that neither a torpedo-like nor an Nrd1-like mechanism functions to terminate transcription of human snRNA genes. Instead, the integrator complex coordinates snRNA 3′-end processing with transcription termination in conjunction with polyadenylation factors. Both Pcf11 and Ssu72 function primarily as transcription terminators rather than RNA 3′-end processing factors for the snRNA genes. In addition, we show that the transcription unit of both U1 and U2 snRNA genes extends to ∼1 kb and is nucleosome-depleted. A negative elongation factor (NELF) involved in the early elongation checkpoint of protein-coding genes (26) co-operates with the other termination factors to ensure that transcription terminates at the end of the nucleosome-depleted region. Interestingly, nucleosome depletion is reversed if the essential snRNA gene-specific initiation factor, proximal sequence element-binding transcription factor (PTF) (also known as SNAPC and PBP) (27–29), is knocked down, indicating that a promoter factor helps define the downstream boundary of the transcription unit.

## MATERIALS AND METHODS

### Chromatin immunoprecipitation analysis

Chromatin immunoprecipitation (ChIP) analysis was carried out as previously described (30). On average, 0.5 × 10^6^ cells were used per immunoprecipitation. Antibodies used in ChIP experiments were purchased from the following manufacturers: anti-pol II (Sigma, N-20), anti H3 (abcam, ab1791), anti-NELF-A (Santa Cruz, sc-32911), anti-Pcf11 (Bethyl, A303-706A), anti-Ssu72 (Santa Cruz, N-16) and anti-Cstf64 (Bethyl, A301-092A). Anti-PTFγ antibodies were a gift from Robert G. Roeder and Jong-Bok Yoon (31). Primer sequences used in ChIP analysis are available on request. Typical quantitative polymerase chain reactions (PCRs) included a 95°C step for 15 min, 40 cycles at 95°C/15 s, 57°C/15 s and 72°C/25 s, followed by a 10-min melt. Percentage input was calculated as (IP average—no antibody average) × 100/input.

### Knockdown experiments

SiRNAs were purchased from Dharmacon. Knockdown experiments were performed in HeLa cells with lipofectAMINE 2000 reagent (Invitrogen) according to the manufacturer’s instructions. Typically, 1 × 10^5^ cells were transfected, in duplicate, with 20 pmoles of the siRNA in a 24-well format. The following day, cells were harvested and re-plated into one well of a 6-well format dish. Twenty-four hours later, cells were transfected again with 100 pmoles of siRNA and harvested for ChIP and western blot analysis the following day.

### Nuclear run-on experiments

Nuclear run-on (NRO) analyses were carried out as described by Cuello *et al.* (32) with 80-nt oligonucleotide probes complementary to RNA transcribed from the U1 and U2 gene. The 3′-ends of the U2 probes correspond to positions −130 (proximal sequence element), +48 (R1), +208 (R2), +288 (R3), +368 (R4), +448 (R5), +528 (R6) and +608 (R7), relative to the site of transcription initiation. The 3′-ends of the U1 probes correspond to positions −120 (proximal sequence element), +83 (N1), +164 (N2), +290 (N3), +375 (N4), +450 (N5), +550 (N6) and +620 (N7), relative to the site of transcription initiation. The 3′-end of the 80-nt 5 S RNA probe corresponds to position +32, relative to the site of transcription initiation. Synthetic U1 and vU1 snRNAs were made by T7 from sequences cloned into pGEM vectors (33).

### RNase protection analysis

Total RNA was prepared using the Trizol (Invitrogen) method, according to manufacturer’s instructions. Samples were DNase treated, phenol/chloroform extracted, precipitated with ethanol and resuspended in water. RNase protection was carried out as outlined in (34).

### Western blot analysis

Antibodies used in western blot studies were purchased from the following manufacturers: anti-SCAF8 (Bethyl, A301-036 A), anti-Senataxin (Bethyl, A301-105 A), anti-Int5 (Bethyl, A301–268 A), anti-Int9 (Bethyl, A300-412 A) and anti-Int11 (abcam, ab75276); anti-Xrn2 was a gift from Natalie Gromak (35).

## RESULTS

### Re-defining the U1 snRNA gene transcription unit

**Figure 1.**
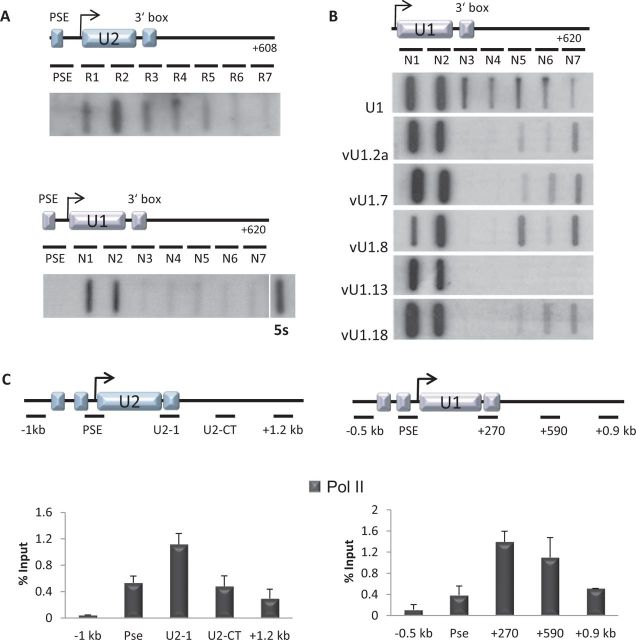
Re-defining the U1 snRNA gene transcription unit. (**A**) Schematics of the U2 and U1 transcription units with the position of the NRO probes are indicated. The proximal sequence element, snRNA encoding and the 3′ box are denoted with blue (U2) and purple (U1) boxes; +608 (U2) and +620 (U1) correspond to the position of the last probe from the first base of the U2 and U1 snRNAs, respectively. The arrow represents the location of the start of transcription. Results of the *in vivo* NRO analysis of the U2 and U1 genes are shown below the schematics. (**B**) Schematic of the U1 snRNA gene labelled as in (A). Results of the NRO analysis with U1 and vU1 snRNA transcripts, generated *in vitro,* hybridized to filters containing probes complementary to the U1 snRNA transcript, as indicated, are shown below the schematic. (**C**) Schematics of the U2 and U1 snRNA genes, labelled as in (A), with the position of the ChIP probes indicated. Graphs represent results of pol II ChIP analysis of the U2 and U1 snRNA genes in HeLa cells with the position of the primers indicated below each graph. Error bars represent standard deviation of at least three independent experiments.

Previous NRO studies indicate that termination of transcription of the U1 snRNA gene occurs immediately downstream of the 3′ box, whereas transcription of the U2 snRNA gene continues for up to 1 kb past the 3′ box (Figure 1A) (32). We have recently shown that in addition to the U1 snRNA gene, there are numerous variant (v)U1 snRNA genes expressed in HeLa cells (33). The vU1 snRNA genes are similar to the U1 snRNA genes in the RNA-encoding region but diverge in sequence immediately downstream of the 3′ box. This is not the case for the U2 snRNA genes, which are within a tandem array of highly conserved 6.1 kb repeats (36). Thus, the marked drop in NRO signal intensity after the U1 snRNA 3′ box could be a consequence of the NRO probes hybridizing to conserved sequences within the RNA-encoding regions of both U1 and vU1 snRNA genes but only to the 3′ flanking regions of the transcripts from the U1 snRNA genes. To test this possibility, RNA corresponding to transcripts from vU1 snRNA genes was generated *in vitro* and hybridized to filters with probes complementary to transcripts from the U1 snRNA genes. The U1 snRNA gene probes cross-hybridize to all vU1 snRNAs analysed within the well-conserved regions, which include the coding and distal 3′ flanking sequences (Figure 1B). Importantly, the probes complementary to the immediate 3′ flanking region of U1 snRNA (N3 and N4) are specific to U1 snRNA genes only, which is consistent with divergence in sequence of all vU1 snRNA genes in this region. This result indicates that the drop in NRO signal intensity immediately downstream from the 3′ box of the U1 snRNA gene likely reflects probe specificity.

To determine more precisely where termination of transcription of the U1 snRNA gene occurs *in vivo,* the density of pol II across the U1 snRNA gene was measured by ChIP analysis with U1 snRNA gene-specific primers. To ensure the primers designed were specific to the U1 snRNA gene only, they were tested in PCR reactions using genomic clones of U1 and vU1.18 and vU1.2 a snRNA genes as template DNA. Genomic (g)DNA was used as a positive control for each primer pair. Only primer pairs specific to U1 snRNA gene give a positive signal in this PCR analysis (Supplementary Figure S1*)*. ChIP analysis with an antibody that recognizes both phosphorylated and unphosphorylated forms of pol II indicates that pol II remains associated with the U1 snRNA gene up to 900 bp from the start of transcription (Figure 1C). The extended pol II profile does not appear to be a consequence of poor resolution of the ChIP assay as the PTF-γ subunit of the PTF complex associates specifically with the U1 snRNA gene promoter region, as expected (Supplementary Figure S2). These data indicate that termination of transcription of the U1 snRNA gene does not occur immediately downstream of the 3′ box as previously suggested (32). Moreover, global run-on sequencing (GRO-seq) data (37) also indicate that pol II transcribes up to 700 bp from the TSS of both the U2 and U1 genes (Supplementary Figure S3). Taken together, these data support the idea that the U1 and U2 transcription units are more similar than previously considered.

### Human snRNA genes do not use an *Nrd1*-like to terminate transcription

**Figure 2.**
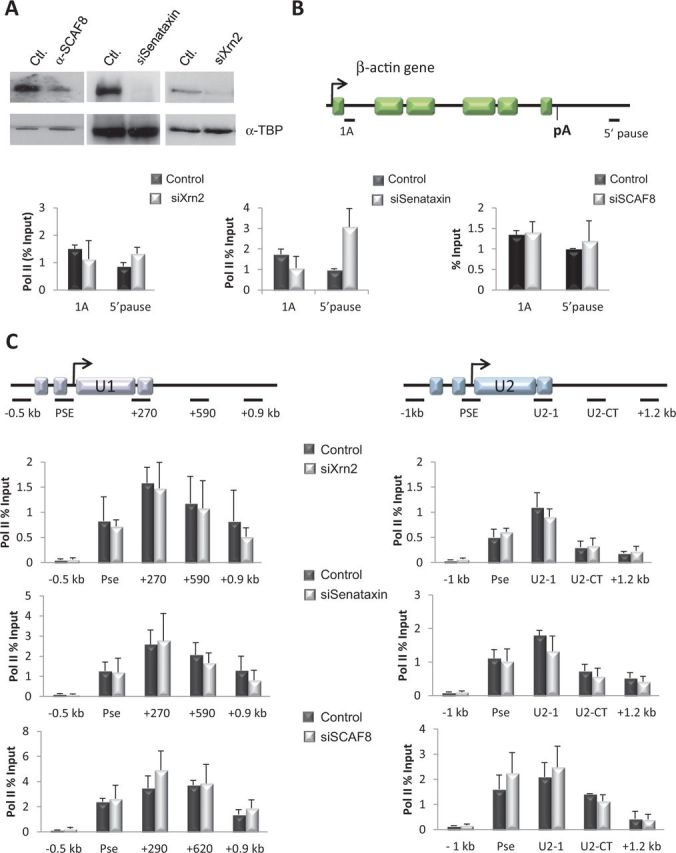
Human snRNA genes do not use an Nrd1-like pathway to terminate transcription *in vivo*. (**A**) Western blot analysis of HeLa whole-cell extracts from control cells or cells transfected with an siRNA specific for SCAF8, senataxin and Xrn2. Antibodies used for detection were α-SCAF8 (left), α-Senataxin (middle) and α-Xrn2 (right). α-TATA-binding protein was used as a control for protein levels. (**B**) Schematic of the β-actin gene with the location of the primers used in ChIP assays indicated. The 5′ pause is described in (39). The arrow represents the TSS, exons are denoted by boxes and the position of the poly A site (pA) is indicated by a vertical line. Graphs represent results of pol II ChIP analysis before and after siRNA-mediated knockdown of Xrn2 (left graph), Senataxin (middle graph) and SCAF8 (right graph). (**C**) Schematics of the U1 and U2 snRNA genes are labelled as in Figure 1. Graphs represent results of pol II ChIP analysis of the U1 (left panel) and U2 (right panel) snRNA genes before and after siRNA-mediated knockdown of Xrn2 (top), Senataxin (middle) and SCAF8 (bottom). Levels of pol II quantitated in (A) and (B), in control and knockdown cells, are normalized to levels quantitated on a non-transcribed region of the genome (120 bp region ∼2 kb upstream of the U2 snRNA gene). Error bars represent standard deviation of at least three independent experiments.

To investigate the mechanism of termination of transcription of the human snRNA genes, we examined the requirement for Xrn2, which is implicated in the ‘torpedo’ mechanism, and Senataxin, which is the human homologue of Sen1. In addition, we also looked at SCAF8 (rA8, RBM16) because it is thought to be a good candidate for the human homologue of Nrd1, as it contains a similar Pol II CTD interacting domain and RNA recognition motif (38). All three factors were knocked down in HeLa cells using specific siRNAs (Figure 2A). Importantly, TATA-binding protein levels remain unchanged after knockdown (Figure 2A). To determine whether these factors play a role in termination, the effect of their knockdown on pol II levels at the end of the snRNA transcription unit was measured by ChIP. The β-actin gene was used as a model for pol II-transcribed protein-coding genes. As expected, reducing the levels of either Xrn2 or Senataxin results in an increase in pol II ChIP signals over the region downstream of the β-actin pA site (Figure 2B [5′ pause], Supplementary Figure S4) (39), whereas pol II association with the U1 and U2 genes remains unchanged (Figure 2C and Supplementary Figure S4). Altering SCAF8 levels has no effect on pol II levels at the end of the β-actin, U1 or U2 genes (Figure 2B and C and Supplementary Figure S4), arguing against a role for this factor in termination of transcription in human cells, although this factor may be redundant with other potential Nrd1 homologues, including SCAF4 (38).

### Integrator subunits couple snRNA 3′-end processing to termination of transcription

**Figure 3.**
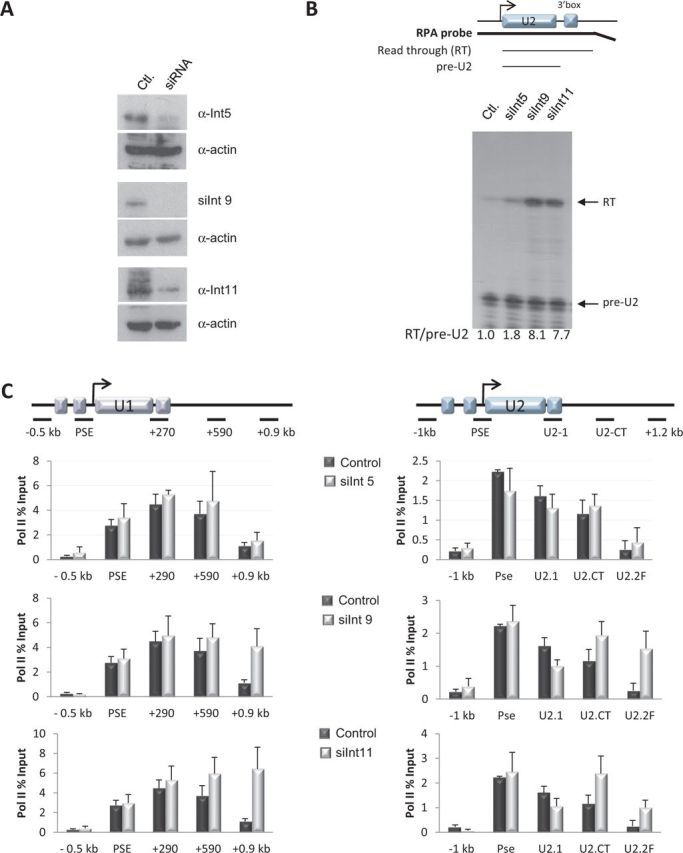
Integrator subunits couple snRNA 3′-end processing to termination of transcription (**A**) Western blot analysis of HeLa whole-cell extracts from control cells or cells transfected with an siRNA specific for Int5, Int9 and Int11. Antibodies used are noted on the right. The α−actin was used as a control for protein levels. (**B**) Schematic of the U2 gene labelled as in Figure 1. Location of the RNase protection analysis (RPA) probe and the expected products of the RPA are noted below the schematic. Results of RPA of RNA transcribed from the endogenous U2 gene in control cells and cells transfected with an siRNA specific for Int5, Int9 and Int11 are shown. Position of the pre-U2 snRNA and unprocessed RT U2 snRNA is noted on the right. The ratio of RT to pre-U2 relative to the control is also noted below each lane. (**C**) Schematics of the U1 and U2 snRNA genes are labelled as in Figure 1. Graphs represent results of pol II ChIP analysis of the U1 (left) and U2 (right) snRNA genes before and after siRNA-mediated knockdown of Int5 (top), Int9 (middle) and Int11 (bottom). Pol II levels between control and knockdown cells were normalized as in Figure 2. Error bars represent standard deviation of at least three independent experiments.

It is well documented that 3′-end processing is tightly coupled to termination of transcription of the pol II-transcribed protein-coding genes, which prompts the question of whether factors involved in 3′-end processing of transcripts from human snRNA genes also influence termination of transcription. An snRNA gene-specific complex, Integrator, is recruited to human snRNA genes to carry out 3′-end processing directed by the conserved 3′ box RNA processing element located within 19 bases downstream of the snRNA-encoding sequence (25). This complex consists of 12 subunits, 10 of which are recruited early in the transcription cycle by interaction with the pol II CTD phosphorylated on Ser7 (40,41). Later in transcription, when the pol II CTD is phosphorylated on both Ser2 and Ser7, a further two subunits, Int9 and Int11, are recruited (42). These factors are thought to be catalytic subunits of the integrator complex, as they are homologues of CPSF73 and CPSF100, respectively, and knockdown of these subunits in both human and *Drosophila* cells abolishes proper 3′-end formation of snRNAs (25,43,44). To determine whether Integrator participates in snRNA gene transcription termination, we targeted the catalytic subunits Int9 and Int11, and the Int5 subunit in knockdown studies using siRNAs. The efficiency of knockdown was assessed by western blot analysis, with α-actin as a loading control (Figure 3A). The results of knockdown (Figure 3B) confirm roles for Int5, Int9 and Int11 in snRNA 3′-end processing, as reducing the level of these factors *in vivo* causes read-through (RT) of the U2 snRNA 3′ box processing element as assessed by RNase protection analysis (a 1.8 -, 8.1 - and 7.7-fold increase in the ratio of RT/pr-U2 snRNA compared with control in the Int5, Int9 and Int11 Kd cells, respectively). These data are consistent with results from a previous study in *Drosophila*, which showed that all Integrator subunits, apart from Int3, Int10 and Int12, participate in snRNA 3′-end processing (44). Pol II ChIP indicates that altering Int5 levels has a small effect on pol II levels at the end of the U1 and U2 snRNA genes, whereas termination of transcription is severely affected following a reduction in Int9 and Int11 levels (Figure 3C and Supplementary Figure S5). These data demonstrate that factors associated with 3′-end processing of transcripts from human snRNA genes are intimately involved in control of termination of transcription.

### mRNA 3′-end processing factors are required for termination of transcription of human snRNA genes

**Figure 4.**
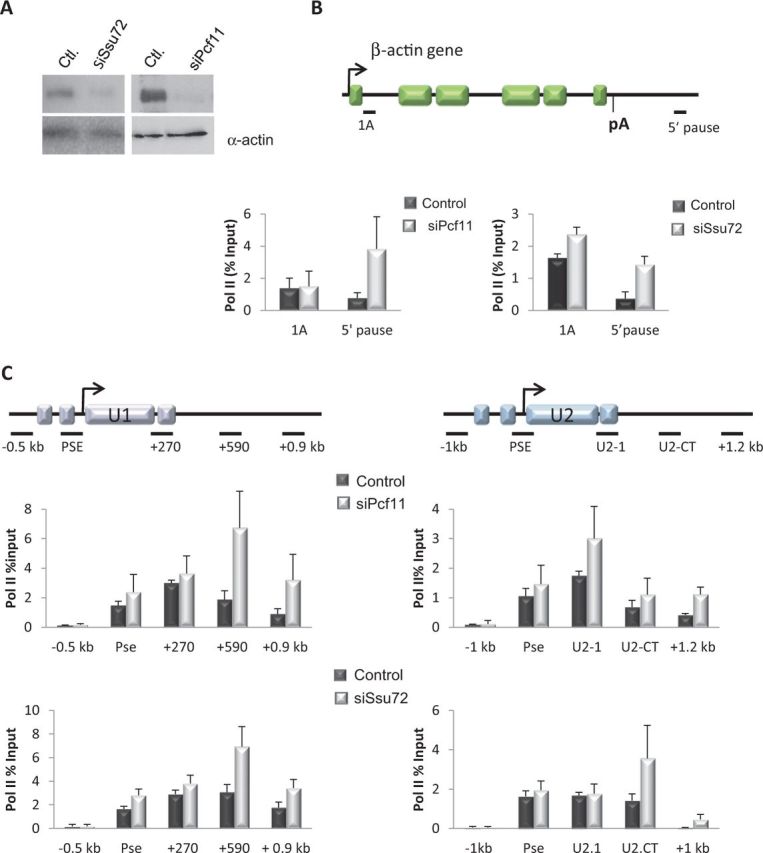
mRNA 3′-end processing factors are required for termination of transcription of human snRNA genes. (**A**) Western blot analysis of HeLa whole-cell extracts from control cells or cells transfected with an siRNA specific for Ssu72 and Pcf11. Antibodies used for detection were α−Ssu72 (left) and α-Pcf11 (right). The α−actin was used as a control for protein levels. (**B**) Schematic of the β-actin gene labelled as in Figure 2. Graphs represent results of pol II ChIP analysis before and after siRNA-mediated knockdown of Pcf11 (left) and Ssu72 (right). (**C**) Schematics of the U1 and U2 snRNA genes are labelled as in Figure 1. Graphs represent results of pol II ChIP analysis of the U1 (left) and U2 (right) snRNA genes before and after siRNA-mediated knockdown of Pcf11 (top) and Ssu72 (bottom). Pol II levels in (A) and (B) were normalized as in Figure 2. Error bars represent standard deviation of at least three independent experiments.

As components of the integrator complex are similar to components of the cleavage/polyadenylation machinery, we wondered whether any mRNA 3′-end processing factors are involved in termination of transcription of human snRNA genes. Previous data indicate that Pcf11 and Ssu72 are required for termination of transcription of both protein-coding and sno-/snRNA genes in yeast (45). Ssu72 is best known for its role as a phospho-Ser5 phosphatase, and more recently as a phospho-Ser7 phosphatase at the ends of mRNA genes (46–49). It also co-purifies with the cleavage and polyadenylation machinery, and mutation of this factor can affect 3′-end processing and/or termination of transcription of pol II-transcribed genes (49–52). To establish whether these factors also participate in human snRNA gene transcription termination, pol II ChIP was carried out after knockdown of Pcf11 or Ssu72. The efficiency of the knockdown was confirmed by western blot analysis (Figure 4A). As expected, depletion of both factors results in an increase of pol II at the end of the β-actin gene (Figure 4B and Supplementary Figure S6). Reducing Pcf11 and Ssu72 also causes an increase in pol II levels at the end of both snRNA genes. (Figure 4C and Supplementary Figure S6). Moreover, ChIP analysis using antibodies to Pcf11 and Ssu72 confirms that these factors are recruited to the snRNA genes (Supplementary Figure S7)*.*

As Pcf11 and Ssu72 are both involved in mRNA 3′-end processing, their requirement for 3′ box-directed 3′-end formation was determined after RNAi-mediated knockdown. As a positive control, cells were treated with the kinase inhibitor KM05382 (KM), which inhibits snRNA 3′-end processing by inhibiting the kinase activity of the CKD9 subunit of the P-TEFb complex (34). There is a marked increase in the unprocessed RT product after treating cells with KM for 2 h (a 52-fold increase in the ratio of RT/pre-U2 snRNA RT compared with control) (Supplementary Figure S8*)*. Cells expressing reduced levels of Pcf11 have a marginal defect in 3′ box-directed processing (a 1.7-fold increase in the ratio of RT/pre-U2 snRNA compared with control), whereas knockdown of Ssu72, Xrn2, SCAF8 or Senataxin has no negative effect on processing. This indicates that Ssu72 is acting only as a termination factor and the effect on termination of transcription is not due to a cleavage defect. Notably, loss of Pcf11, In9 or Int11 leads to similar defects in transcription termination, as assessed by ChIP analysis (compare Figures 3 and 4), whereas reducing Int9 or Int11 levels causes a much bigger defect in snRNA 3′-end processing in comparison with loss of Pcf11 (compare Figure 3 with Supplementary Figure S8). This result suggests that Pcf11 has a more prominent role in termination of transcription of snRNA genes than in RNA 3′-end processing.

Interestingly, Cstf64 is also associated with both protein-coding and snRNA genes (Supplementary Figure S7), raising the possibility that other polyadenylation factors are involved in transcription of snRNA genes and/or processing of the transcripts.

### Chromatin architecture may contribute to termination of transcription of human snRNA genes

**Figure 5.**
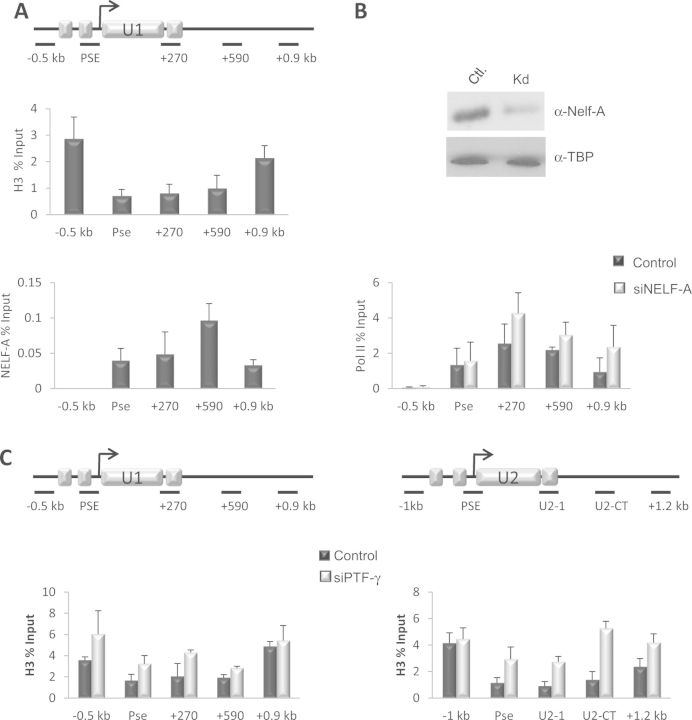
Chromatin architecture may help termination of transcription of human snRNA genes. (**A**) Schematic of the U1 snRNA gene is labelled as in Figure 1. Graphs represent histone H3 (top) and NELF-A (bottom) ChIP analysis of the U1 snRNA gene. (**B**) Western blot analysis of HeLa whole-cell extracts from control cells or cells transfected with an siRNA specific for NELF-A. Antibodies used are noted on the right. The α−TATA-binding protein was used as a control for protein levels. Graph represents results of pol II ChIP analysis of the U1 snRNA gene before and after knockdown of NELF-A. (**C**) Schematics of the U1 and U2 snRNA genes are labelled as in Figure 1. Graphs represent results of histone H3 ChIP analysis of the U1 (left) and U2 (right) snRNA genes before and after siRNA-mediated knockdown of the PTF-γ subunit of the PTF complex. Error bars represent standard deviation of at least three independent experiments.

mRNA genes generally contain a nucleosome-free region that extends to ∼100 nt from the TSS (53,54). The increase of nucleosomes thereafter coincides with the recruitment of two factors involved in early elongation control, the negative elongation complex (NELF-A, -B, -C/D and -E) and the 5,6-dichloro-1-β-D-ribofuranosylbenzimidazole (DRB)-sensitivity factor. Phosphorylation of NELF, DRB-sensitivity factor and the CTD of pol II by CKD9 causes release of NELF and enables pol II to make the transition to productive elongation (26). The U2 snRNA gene has a much more extended nucleosome-free region stretching from the TSS to 1 kb downstream of the coding region, which encompasses the entire transcription unit (55). NELF is recruited to the U2 snRNA gene at the point where the nucleosomes increase, which coincides with the end of the transcription unit. In addition, in expression of snRNA genes, NELF does not function in early transcription elongation control but instead functions as a transcription termination factor, and reduction in NELF levels causes pol II to transcribe past the normal termination point of the human U2 snRNA gene (55). Thus, for the U2 snRNA gene, the chromatin structure, together with association of NELF late in the transcription cycle, may set up a transcriptional roadblock that favours transcription termination over continued elongation. To establish whether pol II encounters an equivalent roadblock during transcription of the U1 snRNA gene, the nucleosome density and NELF levels associated with the U1 snRNA gene were analysed by ChIP with antibodies to histone H3 and the NELF-A subunit. The U1 snRNA gene, like the U2 snRNA gene, has an open chromatin structure that extends across the entire length of the transcription unit (Figure 5A). Similarly, NELF-A recruitment coincides with the increase in H3 levels at the end of the U1 snRNA transcription unit. In addition, NELF also functions as a termination factor for the U1 snRNA genes, as reducing the level of NELF-A causes an increase in the levels of pol II at the end of the U1 snRNA transcription unit (Figure 5A and B).

### PTF is required to maintain an open chromatin structure across the human snRNA genes

Previous work has shown that the open chromatin structure associated with human snRNA genes is transcription- and pol II-independent, as histone profiles remain unchanged following treatment of HeLa cells with α-amanitin, which causes loss of pol II (55). This suggests that a gene-specific factor(s), rather than general factor(s) associated with active transcription, is implicated in modifying the chromatin structure of human snRNA genes. Previous work from our laboratory has also shown that the PTF-γ/SNAP43 subunit of PTF/SNAPc, which is required for snRNA gene expression (56), remains associated with the U2 gene following α-amanitin treatment (42). To test whether PTF is required to maintain nucleosome depletion, PTF-γ was knocked down by RNAi (Supplementary Figure S2). The decrease in PTF-γ occupancy at the promoter is accompanied by an increase in nucleosome density across both the U1 and U2 snRNA genes (Figure 5C). These data suggest that PTF positively influences transcription of snRNA genes by helping remove nucleosomes and/or preventing nucleosome assembly throughout the entire transcription unit. Consequently, the increase in histone density at the end of the U1 and U2 snRNA transcription units, together with the association of NELF-A, could help to promote stalling of the pol II and facilitate termination of transcription.

## DISCUSSION

In yeast, pol II can choose between two distinct pathways to terminate transcription *in vivo*. There is good evidence that the Rat1(Xrn2)-dependent pathway is evolutionarily conserved (13,22,57), but the existence of the Nrd1/Nab3/Sen1 pathway in human cells has not yet been explored. In this report, we show that human snRNA genes do not use an Nrd1-like pathway to terminate their transcription *in vivo* but instead use a distinct mechanism that has more in common with human protein-coding genes than yeast sn/snoRNA genes. In support of this, no Nab3 equivalents have been identified in metazoans. Although SCAF8 has homology to Nrd1 in its CTD-interacting domain and RNA-binding motif (38), our results suggest that this factor does not participate in termination of transcription of human snRNA genes. Consequently, its function in human cells still remains unclear. Although Senataxin and the yeast homologue Sen1p have recently been shown to promote termination of transcription of pol II transcribed genes (39,58), they do not appear to be involved in terminating transcription of the U1 and U2 genes. In contrast, at least two factors required for efficient cleavage and polyadenylation of protein-coding gene transcripts, Pcf11 and Ssu72, are implicated in termination of transcription of the U1 and U2 genes. These data suggest that the mechanism used to terminate transcription of sn/snoRNA genes in yeast has not been conserved to terminate transcription of human snRNA genes.

However, there are notable differences with the termination pathway used by the human snRNA genes to the mechanism used by mRNA genes. Termination of transcription of mRNA genes is closely coupled to 3′-end processing. Cleavage at the poly A site, by CPSF 73, provides an entry site for Xrn2 to degrade the downstream nascent RNA. Additional cleavage and polyadenylation factors including Ssu72, CstF64 and Pcf11 interact with Xrn2 (59) and members of the CPSF complex to couple transcription termination with 3′-end processing. In common with protein-coding genes, termination of transcription of human snRNA genes is dependent on recruitment of factors that are required for cleavage of the nascent transcript to form the 3′ end. However, the CPSF complex is not recruited to human snRNA genes (60,61) but instead a large macromolecular complex, known as Integrator, catalyses cleavage of the nascent pre-snRNA (25). Despite the fact that Integrator cleaves the snRNA ≤19 nt upstream of the 3′-end processing box, creating an entry site for a 5′–3′ exonuclease, there is no evidence that Xrn2 has a function in terminating transcription of human snRNA genes, although we cannot rule out the possibility that an alternative exonuclease is recruited to promote termination via a ‘torpedo-like’ mechanism. Furthermore, neither Ssu72 nor Pcf11 appears to play a major role in snRNA 3′-end formation and instead function primarily as transcription terminator factors for the human snRNA genes. In agreement with this, Pcf11 is capable of dismantling the pol II elongation complex on protein-coding genes, both *in vitro* and *in vivo,* in the absence of RNA 3′-end cleavage (11,12).

Human snRNA genes also use additional gene-specific and general factors to ensure proper transcription termination. In protein-coding genes, pol II travels ≥2 kb after transcription of the polyadenylation site before termination occurs. In the U1 and U2 snRNA genes instead, termination occurs ∼600 bp downstream of the 3′ box. The shorter distance between the 3′ box and termination of transcription may be caused by NELF and the higher level of nucleosomes, which pol II encounters at this point in both the U1 and U2 genes. Pol II may not be able to readily negotiate this block because transcription and recognition of the 3′ box have caused changes to the processivity of pol II because key elongation factors are not recruited to the pol II transcribing these genes, or because NELF/chromatin pauses pol II long enough for the process of termination of transcription to be completed. In accordance with the possibility that NELF serves to pause pol II, knockdown of NELF allows pol II to travel further but termination still occurs within 1 kb of the 3′ box (55). Moreover, recent data have emphasized that nucleosomes act as a barrier to the transcribing polymerase, as the position of pol II pausing after initiation on a chromatin template is determined by the position of the +1 nucleosome (62). PTF-directed clearing of nucleosomes from the transcription unit, therefore, creates an easily traversed template ∼1 kb, with a boundary close to where NELF is recruited. The association of CTCF in the vicinity of NELF in the U2 gene may help to position NELF recruitment and/or the chromatin boundary (55). It is possible that Xrn2 knockdown has no effect because NELF-dependent pausing is sufficient for termination of transcription to occur.

How PTF affects nucleosome occupancy is currently unclear. However, our data are consistent with a previous study implicating PTF in maintaining nucleosome depletion across the U2 gene transcription unit during interphase (63). Persistent binding of PTF, in particular binding of the PTF-γ subunit, to the promoter in metaphase correlates with chromosome fragility of the RNU1/RNU2 loci. In accordance with our data, this may reflect a failure of chromatin condensation owing to the inability of nucleosomes to reform across these multigene loci. PTF may facilitate nucleosome clearing through interaction with chromatin remodelers like CHD8, which was shown to be recruited to the PTF-dependent promoter of the U6 gene (64).

**Figure 6.**
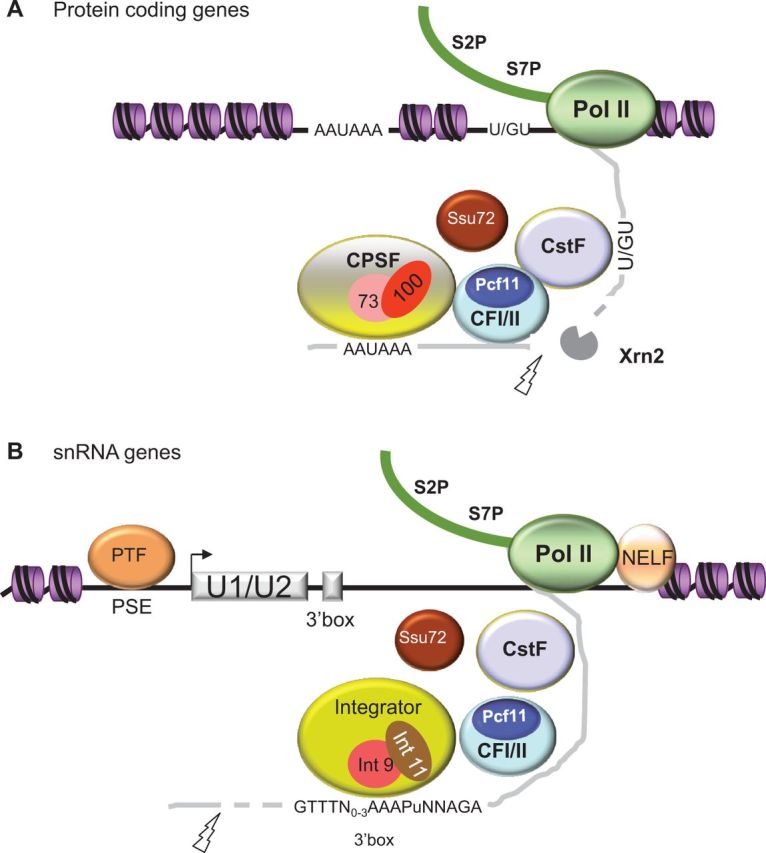
Transcription termination pathways used by pol II transcribing human mRNA and snRNA genes. Termination factors that are common between both genes types are labelled in the same colour. Pol II CTD phosphorylation state is indicated as Phospho-Ser2 (S2P) and Phospho-Ser7 (S7P) (**A**). For protein-coding genes, the CPSF complex and the CstF complex recognize the poly A signal (AAUAAA) and U/GU-rich elements, respectively, as they emerge from the elongation complex. The CPSF-73 subunit, aided by CPSF-100, catalyzes cleavage at the poly A site (indicated by a lightning bolt) creating an entry site for the 5′–3′ exoribonuclease, Xrn2. Xrn2 degrades the downstream RNA product and displaces pol II. The CTD interacting domain of Pcf11 associates with the S2P form of the pol II CTD and bridges the polymerase with the nascent transcript, leading to pol II pausing and dismantling of the elongation complex. (**B**) The gene-specific PTF complex is recruited to the snRNA promoter and clears the transcription unit of nucleosomes to facilitate passage of pol II. The NELF complex is recruited at the end of the nucleosome-depleted region to terminate pol II where nucleosomes are encountered. CPSF-73 is not recruited to human snRNA genes but instead the Integrator complex directs cleavage upstream of the 3′ box processing element (indicated by a lightning bolt). The Int11 subunit is likely to catalyze the cleavage reaction with the help of Int9, as these subunits are homologous to CPSF-73 and CPSF-100, respectively. Thus, Integrator substitutes for the CPSF complex on snRNA genes. Ssu72 and Pcf11 are recruited to snRNA genes but do not have a major function in snRNA 3′-end processing. Instead, Ssu72 and Pcf11 act as transcription terminators for this gene class. CstF64 is also recruited, but its role in snRNA processing and transcription termination is not clear. Xrn2 is not implicated in snRNA transcription termination, but as cleavage upstream of the 3′ box would create an entry site for a 5′–3′ exoribonuclease, we cannot rule out that an alternative enzyme is recruited to promote termination.

Our findings taken together support a model where the major difference between the control of termination of transcription in human protein-coding and snRNA genes may be the use of a different complex to cleave the transcript (Figure 6).

## SUPPLEMENTARY DATA

Supplementary Data are available at NAR Online.

## FUNDING

Wellcome Trust [Grant WT092483 to S.M.] and The Oxford Martin School (to D.O.R.). Funding for open access charge: Wellcome Trust.

*Conflict of interest statement*. None declared.

## Supplementary Material

Supplementary Data
