# Supplementary material for: Human snRNA genes use polyadenylation factors to promote efficient transcription termination
Source: Nucleic Acids Res. 2013 Oct 4;42(1):264–75. doi: 10.1093/nar/gkt892 (PMC3874203; doi:10.1093/nar/gkt892)
Supplement: Supplementary Data [file supp_42_1_264__index.html]

Human snRNA genes use polyadenylation factors to promote efficient transcription termination — Human snRNA genes use polyadenylation factors to promote efficient transcription termination — Supplementary Data 

# Human snRNA genes use polyadenylation factors to promote efficient transcription termination

## Supplementary Data

files

**Files in this Data Supplement:**

- Supplementary Data - pdf file
