# Supplementary material for: Human snRNA genes use polyadenylation factors to promote efficient transcription termination
Source: Nucleic Acids Res. 2013 Oct 4;42(1):264–75. doi: 10.1093/nar/gkt892 (PMC3874203; doi:10.1093/nar/gkt892)
Supplement: Supplementary Data [file supp_gkt892_nar-01786-a-2013-File003.pdf]

Supplementary Material

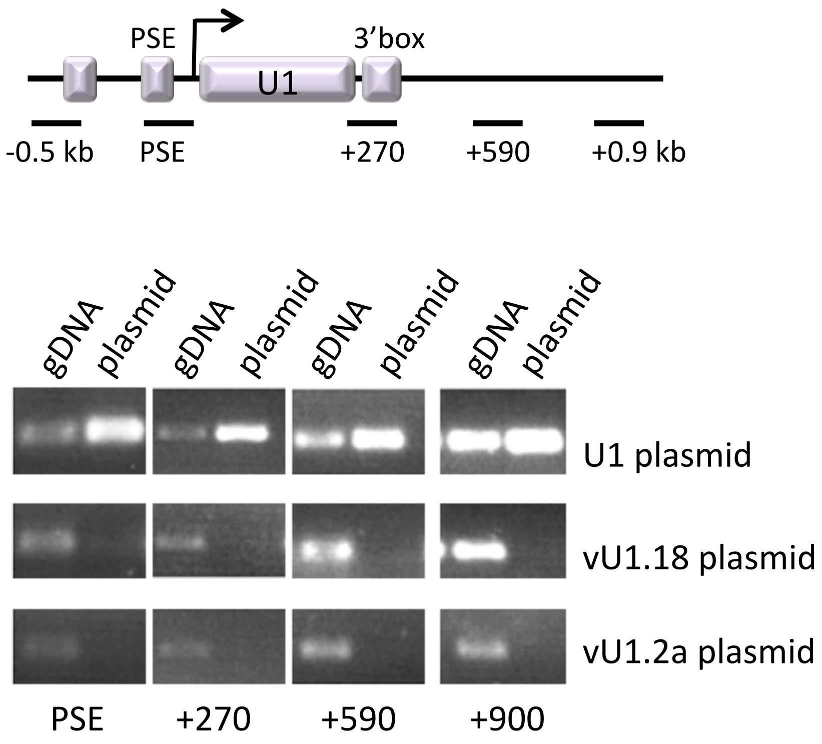

Supplementary Figure 1: Primer specificity controls.
Primers used in ChIP assays were tested for gene specificity against a panel of U1 and vU1 snRNA-containing plasmids. Each plasmid contained genomic sequences, including ~100 bp of the 5′ flanking sequences, the snRNA encoding sequence and 900 bp of the 3′ flanking region, representing the U1 and vU1.2a and vU1.18 snRNA genes. The position of the primers is indicated in the schematic.

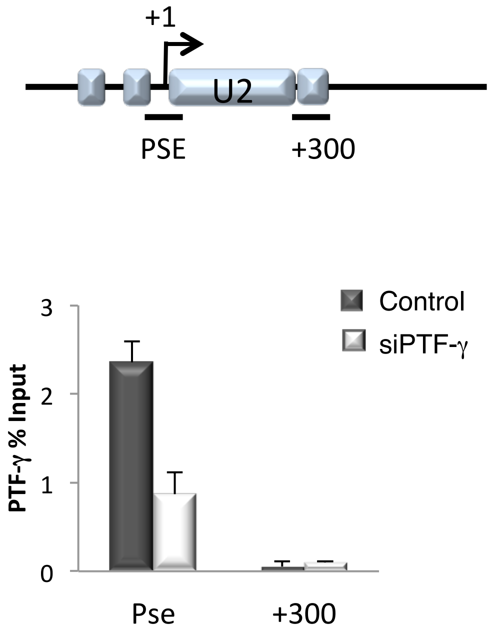

Supplementary Figure 2: Resolution of ChIP analysis
Schematic of the U2 snRNA gene is labelled as in Figure 1. The graph represents the results of PTF-γ ChIP analysis on the U2 snRNA gene. Sonication of the DNA is such that quantification of PTF-γ association is restricted to the PSE region only.

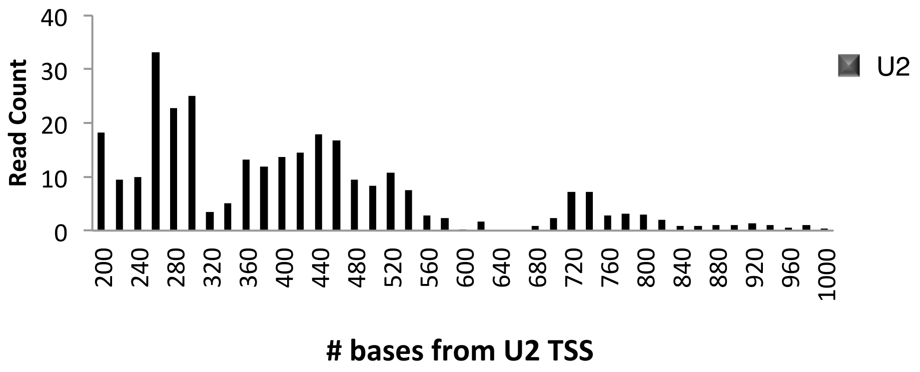

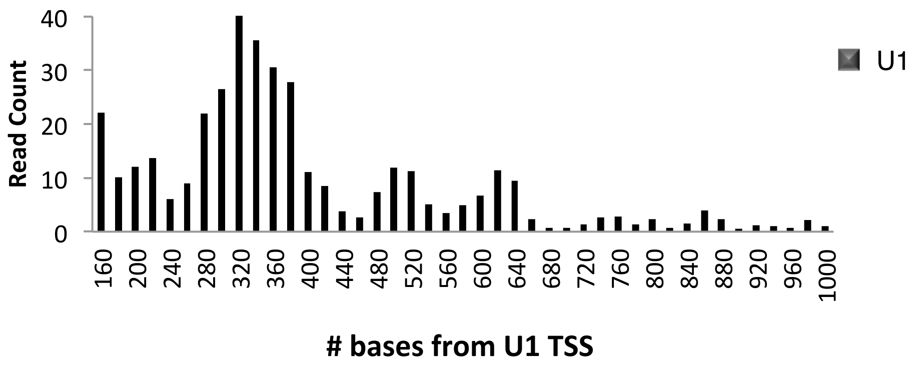

Supplementary Figure 3: The transcription units of the U1 and U2 snRNA genes extend to ~ 1 kb. Graphs represent results of analysis of GRO-seq data (37) for nascent U1 and U2 snRNA sequences. The Y-axes represent read counts at various positions (x-axes) within the 3′ flanking regions of the U2 (Top) and U1 (bottom) snRNA genes.

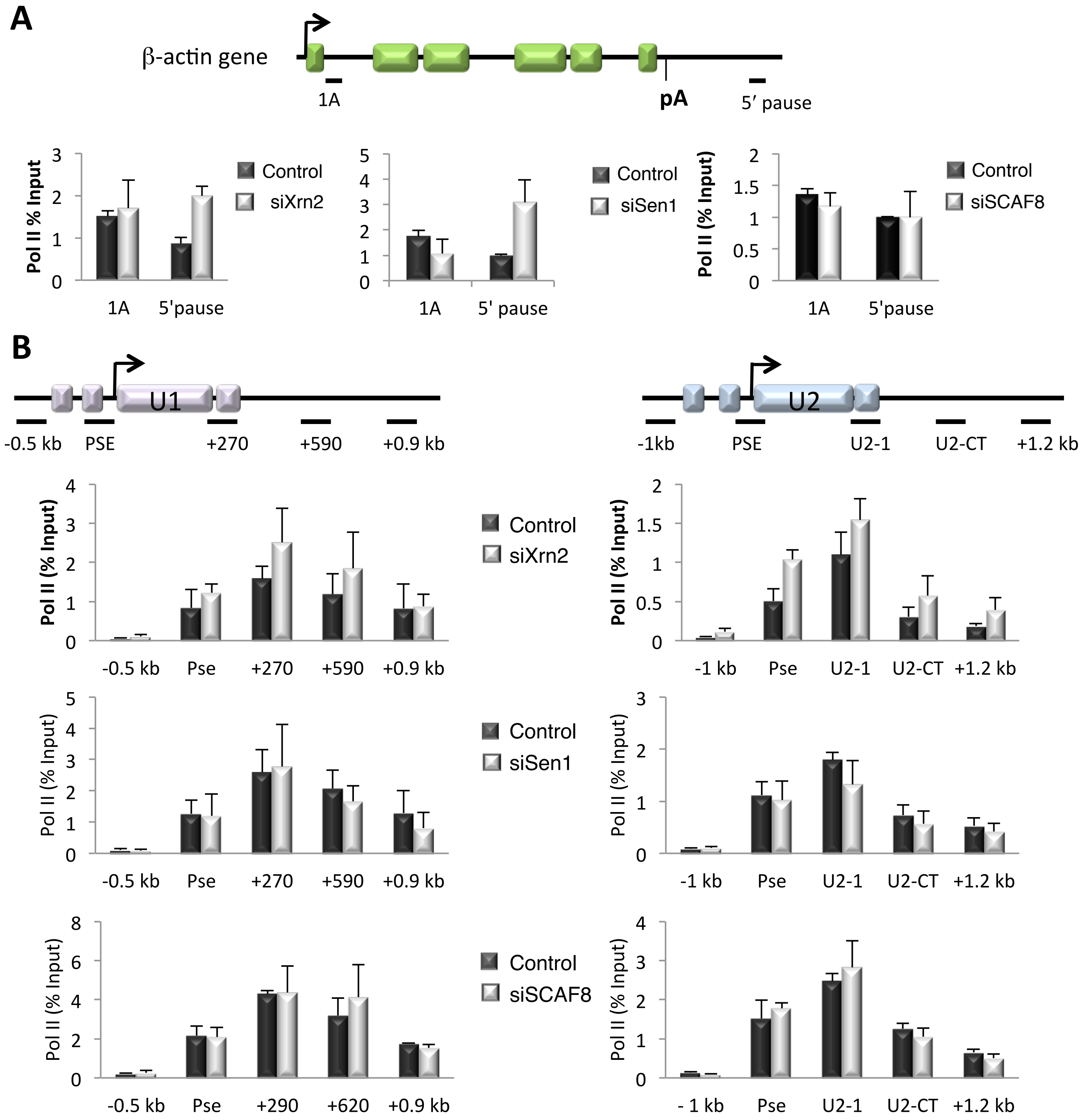

Supplementary Figure 4: Pol II ChIP analysis after Xrn2, Senataxin and SCAF8 knockdown, without normalization.

A) Schematic of the β-actin gene is labelled as in Figure 4. **B**) Graphs represent results of pol II ChIP analysis Before and after siRNA mediated knockdown of Xrn2 (left graph), Senataxin (middle graph) and SCAF8 (right graph) without normalization to the pol II levels associated with a non-transcribed region of the genome (a 120 bp region 2 kb upstream of the U2 snRNA gene). **C)** Schematics of the U1 and U2 snRNA genes are labelled as in Figure 1. Graphs represent results of pol II ChIP analysis of the U1 (left panel) and U2 (right panel) snRNA genes before and after siRNA-mediated knockdown of Xrn2 (top), Senataxin (middle) and SCAF8 (bottom), without normalization to pol II levels associated with a non-transcribed region of the genome. Error bars represent standard deviation of at least 3 independent experiments.

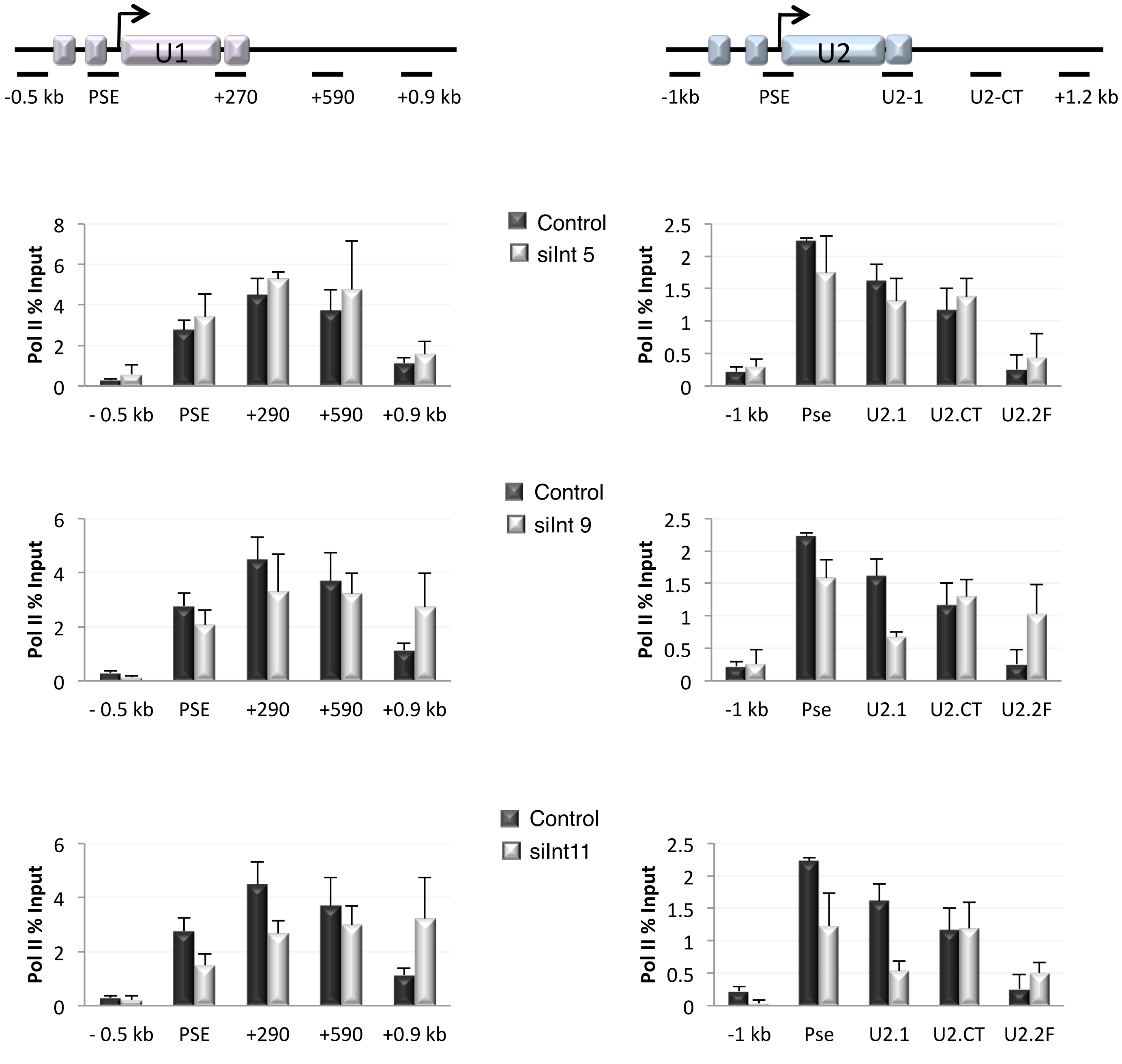

Supplementary Figure 5: Pol II ChIP analysis after Int5, Int9 and Int11 knockdown, without normalization.

Schematics of the U1 and U2 snRNA genes are labelled as in Figure 1. Graphs represent results of pol II ChIP analysis of the U1 (left panel) and U2 (right panel) snRNA genes before and after siRNA-mediated knockdown of Int5 (top), Int9 (middle) and Int11 (bottom), without normalization to pol II levels associated with a non-transcribed region of the genome. Error bars represent standard deviation of at least 3 independent experiments.

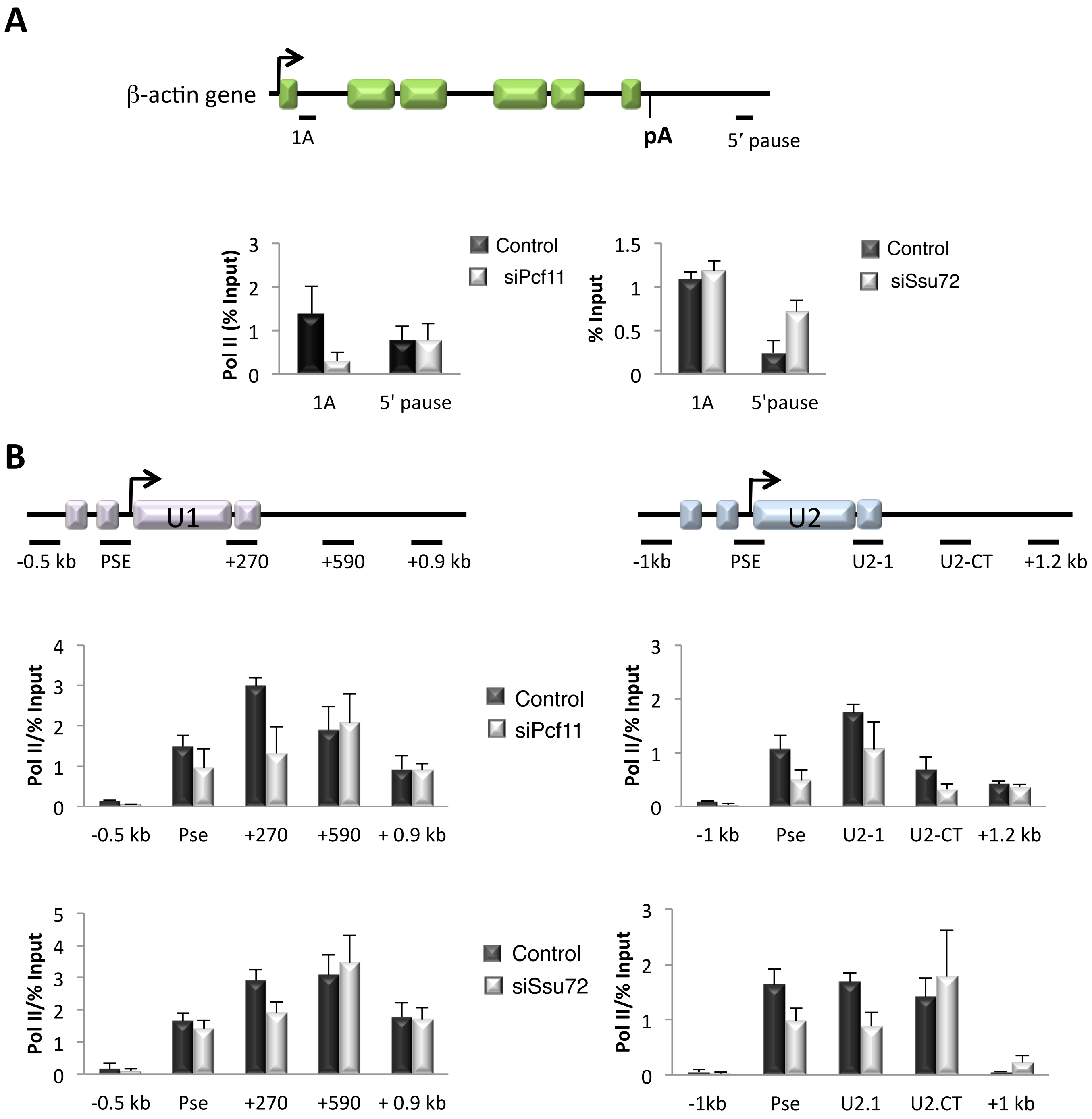

Supplementary Figure 6: Pol II ChIP analysis after Pcf11 and Ssu72 knockdown, without normalization. **A**) Schematic of the β-actin gene labelled as in Figure 2. Graphs represent results of pol II ChIP analysis before and after siRNA-mediated knockdown of Pcf11 (left graph) and Ssu72 (right graph), without normalization to pol II levels associated with a non-transcribed region of the genome. **B)** Schematics of the U1 and U2 snRNA genes are labelled as in Figure 1. Graphs represent results of pol II ChIP analysis of the U1 (left panel) and U2 (right panel) snRNA genes before and after siRNA-mediated knockdown of Pcf11 (top) and Ssu72 (bottom). Pol II levels in (A) and (B), without normalization to pol II levels associated with a non-transcribed region of the genome. Error bars represent standard deviation of at least 3 independent experiments.

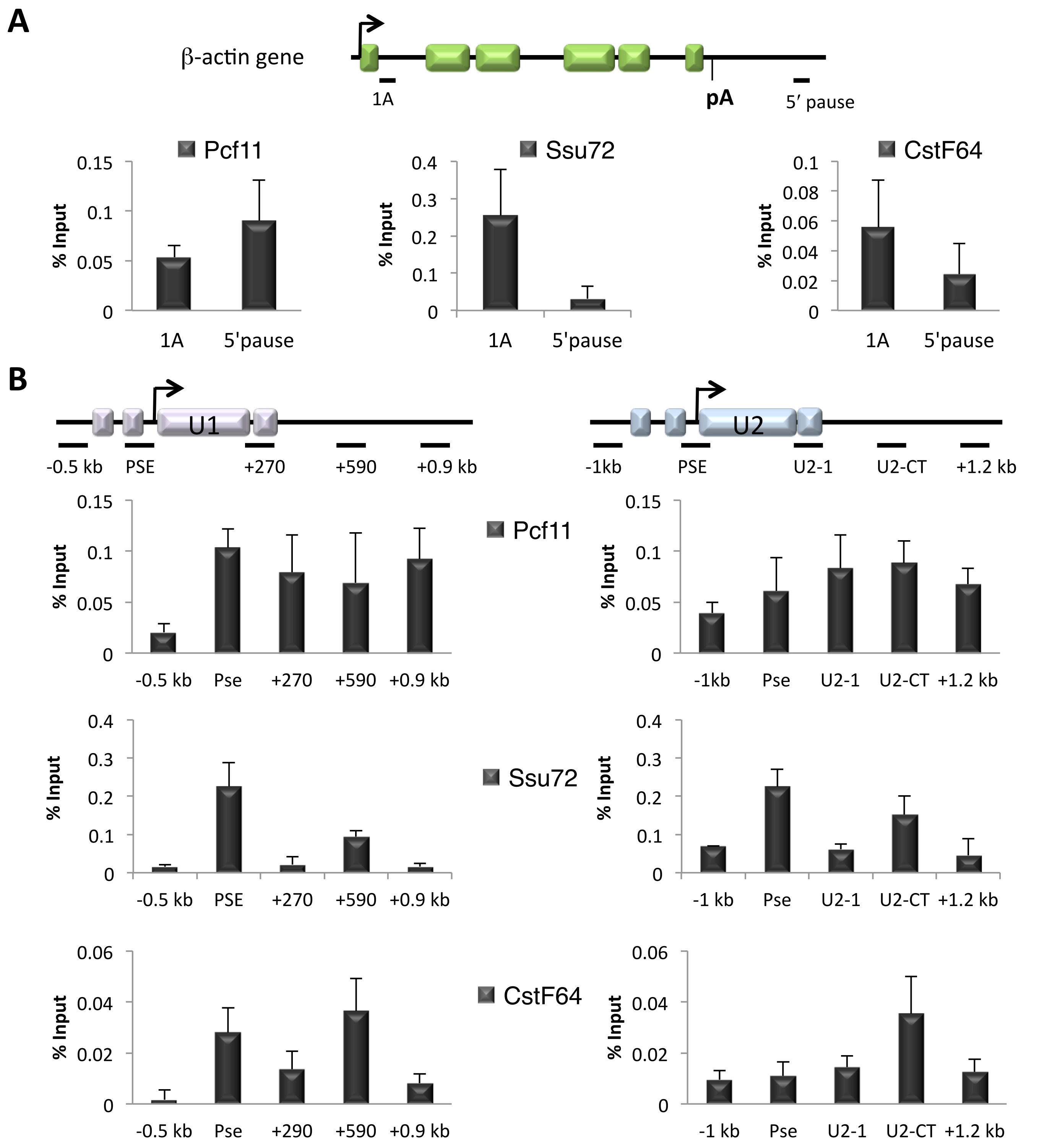

Supplementary Figure 7: ChIP analysis of Pcf11, Ssu72 and CstF64 association with the human β-actin and snRNA genes.

A) Schematic of the β-actin gene labelled as in Figure 2. Graphs represent results of ChIP analysis of the β-actin gene with antibodies targeting Pcf11 (left graph), Ssu72 (middle graph) and CstF64 (right graph). **B)** Schematics of the U1 and U2 snRNA genes labelled as in Figure 2. Graphs represent results of ChIP analysis of the U1 anRNA gene (left panel) and U2 snRNA gene (right panel) with antibodies targeting Pcf11 (top), Ssu72 (middle) and CstF64 (bottom). Error bars represent standard deviation of at least 3 independent experiments.

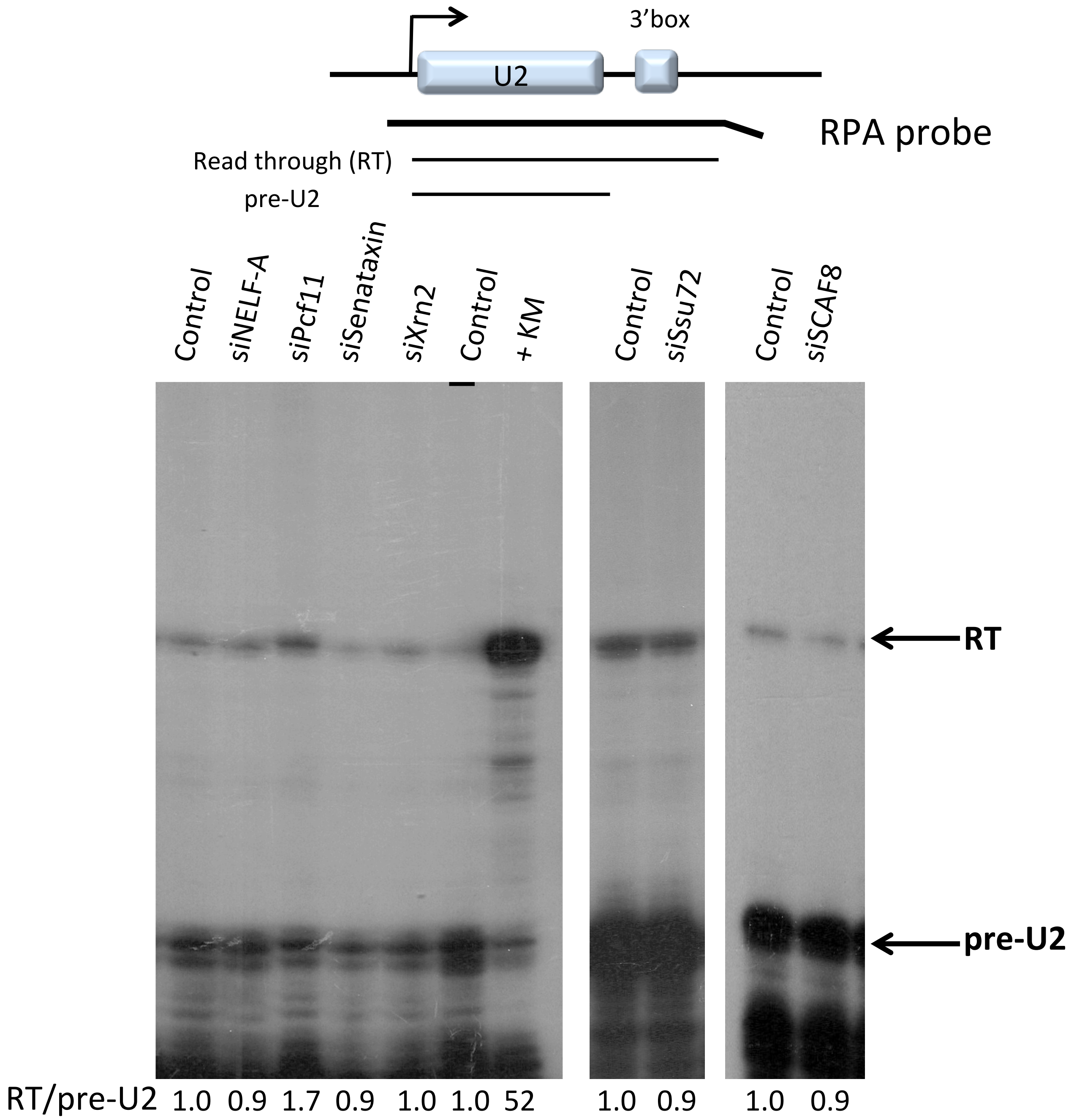

Supplementary Figure 8: mRNA cleavage and polyadenylation factors play a minor role in snRNA 3’end processing.

Schematic of the U2 gene labelled as in Figure 1. Location of the RNase Protection Analysis (RPA) probe and the expected products of the RPA are noted underneath the schematic. Results of RPA of RNA transcribed from the endogenous U2 gene in control cells and cells transfected with siRNAs specific for NELF-A. Pcf11, Senataxin, Xrn2, Ssu72 and SCAF8 are shown. Position of the pre-U2 snRNA and unprocessed read-through (RT) U2 snRNA are noted on the right. The ratio of RT to pre-U2 snRNA Normalised to the control is also noted below each lane. Cells were also treated with 100 uM of KM05382 (KM) for 2 hr as a positive control for U2 snRNA 3’end processing defects.
